# Medication errors with electronic prescribing (eP): Two views of the same picture

**DOI:** 10.1186/1472-6963-10-135

**Published:** 2010-05-24

**Authors:** Imogen Savage, Tony Cornford, Ela Klecun, Nick Barber, Sarah Clifford, Bryony Dean Franklin

**Affiliations:** 1 Department of Practice and Policy, the School of Pharmacy University of London, London, UK; 2 Department of Information Systems, London School of Economics and Political Science, London, UK; 3 Centre for Medication Safety and Service Quality, Imperial College Healthcare NHS Trust, London, UK

## Abstract

**Background:**

Quantitative prospective methods are widely used to evaluate the impact of new technologies such as electronic prescribing (eP) on medication errors. However, they are labour-intensive and it is not always feasible to obtain pre-intervention data. Our objective was to compare the eP medication error picture obtained with retrospective quantitative and qualitative methods.

**Methods:**

The study was carried out at one English district general hospital approximately two years after implementation of an integrated electronic prescribing, administration and records system. *Quantitative: *A structured retrospective analysis was carried out of clinical records and medication orders for 75 randomly selected patients admitted to three wards (medicine, surgery and paediatrics) six months after eP implementation. *Qualitative*: Eight doctors, 6 nurses, 8 pharmacy staff and 4 other staff at senior, middle and junior grades, and 19 adult patients on acute surgical and medical wards were interviewed. Staff interviews explored experiences of developing and working with the system; patient interviews focused on experiences of medicine prescribing and administration on the ward. Interview transcripts were searched systematically for accounts of medication incidents. A classification scheme was developed and applied to the errors identified in the records review.

**Results:**

The two approaches produced similar pictures of the drug use process. Interviews identified types of error identified in the retrospective notes review plus two eP-specific errors which were not detected by record review. Interview data took less time to collect than record review, and provided rich data on the prescribing process, and reasons for delays or non-administration of medicines, including "once only" orders and "as required" medicines.

**Conclusions:**

The qualitative approach provided more understanding of processes, and some insights into why medication errors can happen. The method is cost-effective and could be used to supplement information from anonymous error reporting schemes.

## Background

Replacing inpatient paper medication orders and medical notes with electronic records is widely seen as the key step to improve patient safety both in the United Kingdom [1-3] and elsewhere [4]. Most UK hospital trusts are reported [1] to think that computerisation will reduce medication errors by increasing control over prescribing. However, very few UK hospitals [5,6] have actually implemented hospital-wide electronic systems for ordering and recording administration of medicines (both defined as electronic prescribing in the UK). A similar, though slightly better, situation has been reported in Norway [7] with implementation in most hospitals "stalled". In the USA, widely perceived as leading the field in hospital computerised prescribing, less than 10% of hospitals are reported [8] to have electronic systems.

This is not really surprising as information and communication technology systems are complex and costly interventions. Committing to a particular system requires belief in the evidence that it will be better than current practice, and that potential problems can be managed [9]. Such evidence usually comes from evaluation studies.

The majority of research studies testing the effect of interventions on medication prescribing error rates have used prospective process (observational error counting) or outcome (harm recording) methods [10]. Prospective detection of unintended injury caused by medical management has higher face validity than retrospective methods [11]. For medication errors [12-14] the advantage of prospective methodology is that it allows some or all of the data collection to be done as part of a pharmacist's normal prescription (order) monitoring duties, so reducing research staff workload and cost. The drawback is that if- as frequently happens- an evaluation is commissioned quite close to the time of implementation, it may not be possible for the evaluators to obtain access to the site before the intervention is put in place. In such cases, the only option is to gather pre-intervention data retrospectively, an approach which has been little used in the UK [10,15].

To date, retrospective eP evaluations have involved carrying out a structured clinical review of a sample of patient medical records to identify, classify and count different types of medication errors. However, the people providing or receiving hospital care also offer a rich source of data on the day-to-day workings of a newly implemented eP system. Interviews with stakeholders have already been used to understand the ways such a system can be introduced into, and become part of, hospital practice [15,16].

The aim of this study is to compare the post-eP error pictures provided by retrospective record review and by interviews with staff and patients within one hospital, and to consider the usefulness of interviews as an evaluation method.

## Methods

This retrospective evaluation was approved by the South East Staffordshire local research ethics committee (LREC) and conducted at Queen's Hospital Burton upon Trent [QHB]. This district general hospital was an early adopter of eP and has a long-established system [15] operating as part of a Meditech hospital-wide information system (HIS) and linked to all other electronic patient and laboratory data. Traditional paper medical notes were maintained but all other records were made and stored electronically. Wireless laptops and static terminals allowed access from almost anywhere in the hospital site. Senior staff could also access the system from home.

All authorised staff had access to patient care records; pharmacists used the system to carry out daily prescription review of their allotted patients. System entry was via personal identification number (PIN) and password, issued after mandatory in-house training. All activities were fully auditable.

QHB had a clear policy on eP decision support: the prescriber remained responsible for the decision and hence most of the support was ensuring that drugs prescribed were on the hospital formulary, in doses and formulations that could be supplied. The eP system had been customised to give prescriber "red box" warnings only for clinically significant drug-drug interactions, as defined in the British National Formulary. These could be over-ridden if the prescriber wished, Default doses, dosing frequencies and maximum dose warnings were only set for specific drugs when the consensus was that these would be useful. Drug-allergy and drug-laboratory tests (eg: renal function; INR) checks were not activated.

Implementation began in late 1996 and by 2002, electronic prescribing and administration of medications was the norm on all NHS wards, with paper charts reserved for drugs with highly variable regimens (eg: insulin).

Our data collection began approximately two years after eP rollout was complete. Initial contact with the hospital was made through the head of pharmacy services who then provided information about the study to potential participants. Verbal informed consent was obtained and documented for all interviewees; patients were also given an information leaflet. The LREC did not require patient consent for the retrospective record review.

### Record review data

Patients were sampled from three wards (general medicine; general surgery; paediatrics) which had different versions of the Meditech software introduced at different times between 1999 and 2002. For each ward, data were collected for 25 randomly selected patients admitted in the calendar month six months before implementation of eP and for 25 patients admitted in the calendar month six months afterwards. Only post-eP data will be presented in this paper. Patients were identified from the HIS and all electronic and paper records retrieved.

One research pharmacist carried out a structured review of all medication orders and laboratory tests for each patient throughout his or her stay on the ward. The total number of medication orders written was also recorded. An electronic retrospective review form (RRF) adapted from that used in the first UK study of iatrogenic injury [17] was used to capture data for each patient reviewed, and to classify errors according to published criteria [18]. A short text description providing information on the drug, dose and clinical situation was recorded for each error identified.

### Interview data

Pharmacy staff and the majority of senior clinicians were identified and approached by a senior hospital manager. Other clinical and ward staff were recruited by senior pharmacists responsible for the relevant wards or identified by the research team during interviews and department visits. A staff announcement was also put out on the HIS.

Twenty-six staff (8 doctors, 6 nurses, 8 pharmacists/technicians and 4 others) at senior, middle and junior grades, and 19 adult in-patients were interviewed. All staff participants used the HIS as part of their work. The majority of interviews were tape-recorded. If recording was not feasible (because of the setting), written notes were made.

The interview topic guide [15] was developed from initial interviews with four members of the implementation team and three users (prescribers). There were no direct questions about medication errors. All staff interviews explored stakeholder views of key benefits and problems, and influences on working practices. Questions were tailored to the respondent's professional group.

Adult patients were recruited from all general surgery and acute medicine wards in the hospital, including the two wards used in the record review. Recruiting children was not attempted. Nineteen men and women aged 26 to over 70 took part. Sampling was purposive, based on use of "*as required" *medication and previous admission history. Using Meditech, a senior pharmacist generated a paper listing of eligible patients for each ward. The researchers then checked with the ward sister to determine if patients were available, and well enough to be approached. Interviews were shorter than for staff and focused on the system for prescribing and administering medicines on the ward [15]. We asked specific questions about missed or refused doses, access to medicines outside normal drug round times, and discharge medication.

### Data analysis

The text descriptions of errors found in the retrospective record review were entered into the database by the research pharmacist and reviewed by BDF.

Interview data were collected and analysed by TC, EK, IS and SC. Transcripts and notes were read independently, then emerging themes discussed and agreed. Interview transcripts were then searched systematically for accounts of medication incidents, both actual and hypothetical. A classification scheme based on the stage of the medicine use process was developed through iterative review of individual reports [19] and then applied to the text descriptions of errors identified in the records review.

The manpower required to collect data for each approach was estimated from staff diaries at the end of the study.

## Results

The record review database included 85 individual descriptions of prescribing and administration errors, Three of these (one drug-drug interaction and two failures to adjust dose in renal impairment) resulted in harm [15].

Seventeen of the 26 staff interviews contained 69 accounts of possible medication errors. One of these accounts concerned fluid overload with intravenous therapy and had serious consequences. Three of the 19 patient interviews also contained error accounts. Nine staff did not describe possible medication errors. Four of them had no direct involvement with medication, three were nurses, and two were senior members of the original implementation team.

**Figure 1:**
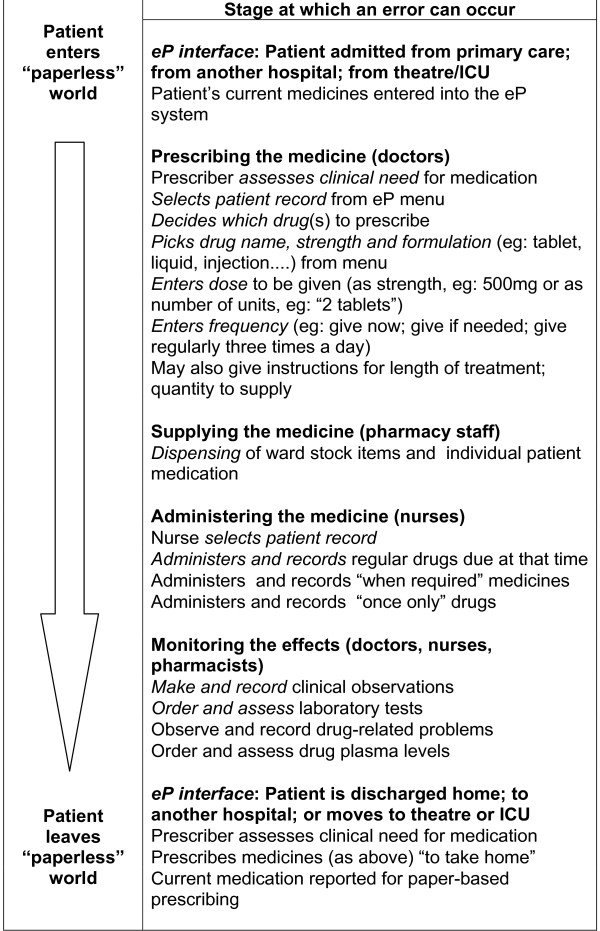
**Error classification scheme developed from staff interviews**.

Figure 1 shows the classification scheme developed from the staff interviews. Table 1 presents the types of error described by different respondent groups. Table 2 compares the types of error identified using interviews and with record review.

**Table 1:** Number of accounts of different types of medication errors described in staff and patient interviews.

Data source	eP interface	Prescribing	Supply	Administration	Monitoring
**Doctors (7)**	1	19	0	8	5
**Nurses (3)**	0	3	0	7	1
**Pharmacy staff (7)**	4	15	1	4	1
**Patients (3)**	1	1	0	1	0
**TOTAL**	6	38	1	20	7

**Table 2:** Medication errors identified using interviews and record review.

Type of medication error	Was error identified using this method?	
	Record review	Interview
**eP interface**	yes	yes
		
**Prescribing**		
Clinical need	yes	yes
Patient record selection	no	yes
Drug choice	yes	yes
Menu picking	no	yes
Dose, form and dosing frequency	yes	yes
Drug/dose duplication	yes	yes
		
**Supplying**	yes	yes
		
**Administering**		
Dose omission (regular medication)	yes	yes
Dose omission (once only or if required)	no	yes
Recording of administered dose	yes	yes
		
**Monitoring**	yes	yes

Details of specific errors identified are set out below, ordered under the headings used in Figure 1. This scheme follows the drug use process from the decision to prescribe through to administering, recording and monitoring the effects of medication, and reflects the way that subjects "talked through the screens" as they explained how they used the eP system.

### eP interface errors

At QHB, the vast majority of clinical information was available electronically through the HIS. Outside the hospital, the situation was different. Patients admitted to and discharged from wards using eP therefore moved across an interface between a mostly paper-free and mostly paper-based world. As they moved, errors could occur.

One staff interview described a case where medicines had been omitted from a patient's prescription on admission. The patient had been in hospital for two months before she told the pharmacist that she normally used eye-drops at home. A patient recounted a similar incident. In another interview, a junior doctor spoke of a diabetic patient who had been discharged to a care home with no insulin because the prescriber had not put a note on the eP system to prescribe this on discharge. Record review found three cases where medication (salbutamol, amitriptyline, insulin) had not been continued as expected when the patient moved between health care sectors. This could happen because medication required for discharge had to be flagged on the system by the prescriber, then "converted" from inpatient orders in the pharmacy before dispensing.

### Errors at the prescribing stage

#### Clinical need

Record review provided a wide range of examples where the need for treatment, review or investigation had not been met. Cases included antibiotic therapy for methicillin-resistant *Staphylococcus aureus *not being prescribed; lack of potassium supplementation in a hypokalaemic asthmatic; not reviewing fluoxetine treatment when hyponatraemia was reported; and untreated hyperlipidaemia. Interviews provided only two examples: neglecting to prescribe antibiotics for a patient with heart valve disease and an account from a patient who reported not being able to get the hospital doctors to prescribe her usual migraine medication.

#### Patient selection

A pharmacy interview described a case where medicines had been prescribed for the wrong patient because of a scrolling error on the patient selection screen. Junior doctors also described near-misses, and said that this error could happen "*because you are not going to the bedside"*. No cases of prescribing to the wrong patient were detected in the record review

#### Drug choice

Both methods identified knowledge-based errors. In record reviews these included potentially clinically significant drug interactions involving aminophylline and phenytoin; use of digoxin and acitretin in renal impairment; and prescribing a penicillin to a patient recorded as possibly allergic. Interviews with doctors mentioned lack of penicillin allergy warnings, and also prescribing a non-steroidal analgesic drug which was "hidden" in a post-operative "order set" to an asthmatic patient. However there were no accounts of drug-drug interactions.

#### Menu picking

The eP prescribing (order entry) screen had "look-up" functions for drug names, doses and routes, and links to drug monographs and local guidelines and policies. In record review it was not possible to say whether picking the wrong drug or strength was purely a scrolling error, or if the prescriber did not know what the correct choice should be. However, pharmacy staff describing cases where doctors had ordered the next drug on the lookup menu considered it very unlikely that this would have happened if the prescription had been hand-written as the drugs (methotrexate instead of methotrimeprazine; ethamsylate instead of ethambutol) were so very different.

#### Drug/dose duplication

Both methods identified unintentional drug duplication (eg: prescribing paracetamol, and a paracetamol-containing combination product) and dose duplication, where a drug (eg: an analgesic or anti-emetic) was prescribed orally and by other routes to allow nurses flexibility depending on the patient condition but the maximum daily dose for the drug was not specified.

#### Dose, form and frequency

Wrong doses, product strengths and dosing frequencies were identified in both interviews and notes review. However interviews primarily cited warfarin and specialist paediatric drugs while errors identified in reviews involved different paediatric and anticoagulant drugs, plus anti-epileptics. Dose frequency errors often involved the lack of a daily limit on "as required" analgesics and anti-emetics, reflecting the inflexibility of eP for this type of drug order. Interviews generated two examples of possible dosing frequency errors: a once-weekly drug prescribed once a day, and drugs for Parkinson's disease. Formulation selection errors mainly involved enteric coated products.

### Supply and administration errors

New orders for drugs not carried as ward stock were picked up by the pharmacy system. Review of administration records identified several regular drugs (including antibiotics and anti-epileptics) which had not been given because no supply was available on the ward. One pharmacy technician described how an incorrect strength of an unidentified drug had been issued as "ward stock". The error had been detected by a nurse during a drug round. There were no accounts of scrolling errors when nurses selected patient names from the screen during drug rounds.

#### Dose omission

The patient drug administration screen listed all regular medication before as required and once-only orders. In interviews, junior doctors and nurses mentioned that urgent once-only orders (eg intravenous furosemide) and some as-required medicines were not given because they "*fell off the screen*" ie could not be seen without "paging down". One patient complained that her once-only dose of an IV antibiotic was several hours overdue. Record review did not identify any errors of this type. We also found some patients who didn't know that they had been prescribed as-required drugs that they could have asked for if needed. Retrospective record review could not identify errors with this type of drug order as it was impossible to know if need had in fact existed.

Scheduling of doses was done at the point of prescribing. Regular medicines were allocated times corresponding to ward drug rounds, with a 2-hour window to allow for variation. Junior doctors and nurses described how default timings in the eP dose scheduling system could delay the start of a new regular medication. Pharmacists and doctors also described difficulties with flexibly dosed drugs such as warfarin and insulin, which required paper records.

### Recording and monitoring errors

#### eP recording

The risk of dose duplication when eP records were not available was identified in both interviews and record review. Nurse and doctor interviews described being unsure, when the system was down, if a patient asking for analgesia had recently received a dose. There was also a report that analgesic doses given in theatre (which did not use eP) might be repeated on the ward. Record review found cases where analgesics and propranolol had been prescribed as regular drugs, but no administration records had been made so it was not clear whether or not they had been given.

Two interviews described cases where medication (topicals and IV fluids) had been given but had not been prescribed on the system and so an eP administration record could not be made. Record review identified several cases where oxygen had been given but not prescribed.

#### Monitoring

Record review identified errors involving lack of renal function monitoring for patients prescribed digoxin, and lack of aminophylline and phenytoin plasma level measurement for patients co-prescribed clarithromycin. Interviews generated possible incidents where warfarin and gentamicin doses had not been adjusted on the basis of test results (eg with warfarin, gentamicin, sliding scale insulin), or where physiological monitoring had not been done (intravenous fluids)

#### Research resources comparison

From initial scoping visit to final interview, collecting data for this study took 14 months. *Record review*: On average, only four patients could be reviewed each day. Despite most records being electronic, retrieving patient information was not particularly quick as there were problems accessing the optical disk archive at busy times. Reviewing 75 patients took approximately 20 researcher-days.

#### Interviews

The 26 staff interviews took approximately 11 researcher-days, with the majority (19) conducted by three researchers over three consecutive days. Patients were recruited and interviewed by two researchers over a second three-day period.

Both record review and interview datasets required further work after collection. Unfortunately diary information was not available for all researchers involved so we were unable to make a comparison.

## Discussion

Our classification of the prescribing process is an oversimplification, developed from the way the staff participants described how they worked. The actual prescribing process is probably much less linear as each step may need to draw on knowledge of previous events (medicines, diagnoses, tests, observations) recorded in patients' charts and notes. The interview sample size was methodologically adequate [19] but the relatively small number of records reviewed was a pragmatic choice, based on previous experience [15]. By its nature, record review is more likely to identify errors of omission than an interview.

Despite these limitations, record review and interviews produced similar pictures of medication errors, although interviewees often described hypothetical incidents without mentioning the specific drugs involved. The three drugs they mentioned most often were warfarin, insulin and intravenous fluids. This is probably because these variable dose medicines were less straightforward to prescribe, with or without eP.

In interviews, the types of incident described reflected the respondent's role. Junior doctors (who do most of the prescribing) and pharmacy staff (who supply the drugs and review most of the orders) described the widest range of incidents. Nurses focused on drug administration and were generally cautious in their responses. All three professional groups had been involved in system development, but nurses were clearly the most concerned about personal accountability [20,21]. Some patients were also concerned about sounding negative, despite assurances that what they told the interviewer would not affect their future care.

The majority of incidents described by staff were close analogies of those that also happen with paper charts. In interviews the inability to "*see the whole picture*" on the screen appeared to be mainly a problem for those relatively new to the system, but much less of a problem than in the recent USA study [8].

The general view is that eP will reduce errors but new technology can also introduce new types of error [8,13,22]. Two of the three serious post-eP errors described by Shulman *et al *[22] were "menu picking" errors. Our retrospective review did not detect these eP-specific errors but, significantly, our interview data did. It also provided rich detail on the technical work [23] of prescribing and administering drugs on the wards.

Robust evaluations are vital tools for persuading users to adopt new technologies, but prospective before-after data collection can take 18 months. This time may not be available, particularly if the evaluation is commissioned around the time of implementation. This can weaken the impact of the findings. The evaluation report [24] of the three pilot sites for the proposed NHS Electronic Health Record (EHR) noted that the study was "seriously hampered" by the failure to build in evaluation before the EHR project started.

The retrospective record review reported here was quicker than the prospective evaluation we did at a different site [12] but record data collection took more researcher-days than interview data. Interviews tailored to address policy-makers' questions could therefore offer good research value particularly if the study site is remote from the research contractors' usual workplace.

Evaluation increases our understanding of the influence of technologies on working practices and patient outcomes, including occurrences of medication errors, but qualitative data on staff perception of errors has been claimed to provide weaker evidence than counts of actual errors [25]. Counts provide error frequencies which are good for efficacy testing but we suggest that counting is not the only way. We need to explore how people change and are changed by systems [7] and use beliefs about errors [8] to maintain and develop safe and effective healthcare working practices.

For example, pharmacists using eP may believe that ready access to a wide variety of computerised results improves their ability to trap prescribing errors, but it may also reduce face-to-face contact with staff and patients because they no longer need to visit the wards to get the clinical information they need for effective prescription review. This change in practice could have a significant effect on inter-professional communication; it might also change the pattern of errors that pharmacists miss.

## Conclusions

Medication error is a sensitive subject, and people may have concerns about reporting actual errors. In our staff interviews, talking about the perceived benefits and drawbacks of their hospital eP system generated many accounts of specific errors which "could" or "might" happen, as well as descriptions of near misses which had been made by other people.

Importantly, these interviews identified the same types of medication errors as did the more resource-intensive record review method. This method therefore offers not only an evaluation tool but also another way of auditing current medication safety problems, which could be used to supplement information from anonymous reporting schemes.

## Competing interests

The authors declare that they have no competing interests.

## Authors' contributions

The work reported in this paper formed part of a larger evaluation, conceived, designed and co-ordinated by NB. IS co-ordinated the qualitative data collection, designed the qualitative/quantitative data comparison, and drafted the manuscript. Interview data were collected and analysed by TC EK IS and SC. BDF designed, co-ordinated and validated the quantitative data collection. All authors read and approved the final manuscript.

## Pre-publication history

The pre-publication history for this paper can be accessed here:

http://www.biomedcentral.com/1472-6963/10/135/prepub
